# Inflammation and Oxidative Stress in Male Infertility: A Narrative Review

**DOI:** 10.1002/rmb2.70090

**Published:** 2026-08-17

**Authors:** Donya Pourbagher, Rastin Bakhtiari Lafmejani, Parham Namdar, Arya Akhavan, Alireza Nikfarjam, Ali Soleimanzadeh

**Affiliations:** ^1^ Department of Theriogenology, Faculty of Veterinary Medicine Urmia University Urmia Iran

**Keywords:** antisperm antibodies, cytokines, inflammation, male infertility, oxidative stress

## Abstract

**Background:**

Male infertility affects 8%–15% of couples, with male factors implicated in about half of cases; many remain idiopathic despite improved diagnostics. This narrative review addresses this gap by examining chronic inflammation as an underappreciated driver of sperm dysfunction that forms a bidirectional, self‐reinforcing cycle with oxidative stress (OS).

**Methods:**

We synthesized studies from 2015 to 2025 identified via PubMed, Scopus, and Web of Science, examining links between inflammation and OS, key immune pathways, and related risk factors through qualitative narrative synthesis.

**Results:**

Inflammation drives leukocyte infiltration, blood‐testis‐barrier breakdown, antisperm‐antibody production, and inflammasome activation, mechanisms that amplify OS and cause sperm DNA damage, impaired motility, apoptosis, and poor semen quality. Intrinsic factors (prostatitis, infection, varicocele) and extrinsic factors (obesity, aging, psychological stress) sustain this cycle through both ROS‐dependent and ROS‐independent, immune‐mediated pathways. Paternal inflammation may also induce epigenetic changes in sperm with potential consequences for offspring health.

**Conclusion:**

Antioxidant monotherapy alone is often insufficient when an underlying immune driver persists. Immune‐focused diagnostics combined with individualized, mechanism‐based treatment strategies may improve personalized care and fertility outcomes.

**Abbreviations**
ART: assisted reproductive technology
ASAs: antisperm antibodies
BPA: bisphenol A
BTB: blood‐testis barrier
CP/CPPS: chronic prostatitis/chronic pelvic pain syndrome
DEHP: di‐(2‐ethylhexyl) phthalate
HLA: human leukocyte antigen
HPA: hypothalamic–pituitary–adrenal
IFN‐γ: interferon‐gamma
IL‐10: interleukin‐10
IL‐1β: interleukin‐1 beta
IL‐6: interleukin‐6
MAGI: male accessory gland infections
MDA: malondialdehyde
NSAIDs: nonsteroidal anti‐inflammatory drugs
OS: oxidative stress
ROS: reactive oxygen species
TGF‐β: transforming growth factor‐beta
TNF‐α: tumor necrosis factor‐alpha

## 1 Introduction

Male infertility is conventionally defined by the World Health Organization as the inability of a man to impregnate a fertile female partner after at least 12 months of regular, unprotected sexual intercourse [1]. This condition represents a growing problem for couples who are considered reproductive all over the world and has definitely led to medical and psychological burdens for these individuals. Whereas estimates of the prevalence of infertility vary depending on geographical locations and research methodologies, approximately 8%–15% of all couples are affected, with male factors accounting for about half of all cases (either singly or in combination with female ones) [2]. Although diagnostic methods have improved, many men are still given a diagnosis of “idiopathic infertility.” This highlights how far our knowledge still is from completely understanding the mechanisms, across the whole male reproductive system, that interfere with spermatogenesis and sperm function [3]. Clinically, male infertility may manifest as abnormalities in any of the following: sperm number, motility, or morphology; capacitation or acrosome reaction; or immune functions, such as antisperm antibodies [4]. Semen quality reflects not only reproductive health but also overall male health, including cardiometabolic disease and the long‐term risks of some cancers [5, 6].

There is growing support for placing inflammation as a central driver of sperm dysfunction and impaired spermatogenesis in many pathways to male infertility. However, it has not historically got the attention it deserves in either research or clinical practice. Inflammation in the male reproductive tract can be acute and evident, as with orchitis or epididymitis, but it more often takes a subtle yet chronic course [7]. Acute inflammatory episodes (e.g., orchitis, epididymitis) mobilize immune cells, surge pro‐inflammatory cytokines, and create spikes in oxidative stress that may acutely compromise sperm function and, in extreme cases, lead to chronic tissue damage if inadequately resolved [8]. Chronic inflammation is gradual but equally destructive; it often remains clinically undetected, as moderate but sustained elevations in cytokines and leukocytes slowly erode the seminiferous epithelium, decrease sperm production, disrupt epididymal maturation, and increase DNA fragmentation [9, 10]. Because these subclinical inflammatory changes are rarely captured by a standard semen analysis, men with seemingly normal sperm parameters may still have underlying inflammation that disrupts fertility.

Understanding the unique immune environments of the testes, epididymis, and seminal plasma is essential to understanding how inflammation degrades fertility. The blood‐testis barrier (BTB) shields postmeiotic germ cells from the general immune system [11]. Anti‐inflammatory factors such as transforming growth factor‐beta (TGF‐β) and interleukin‐10 (IL‐10), along with resident testicular macrophages, interact to maintain this balance [9]. Infections, trauma, varicocele, surgical procedures, and environmental toxins can all compromise BTB or epididymal barrier integrity, thereby exposing sequestered postmeiotic sperm‐specific antigens (which the immune system has never previously encountered) and triggering an exaggerated immune response. Such a response may be characterized by the production of antisperm antibodies, activation of complement cascades, deposition of immune complexes, and chronic inflammation that remodels testicular architecture [4]. Even transient disruptions can have long‐lasting consequences; antibodies produced in a single event may persist, weakening motility, inducing agglutination, and blocking fertilization long after the triggering event has resolved [4].

The relationship between inflammation and oxidative stress (OS) is closely intertwined. Although moderate levels of reactive oxygen species (ROS) are important for physiological processes, such as capacitation and the acrosome reaction, excessive ROS causes havoc via lipid peroxidation, protein damage, mitochondrial dysfunction, and DNA breaks. OS has been implicated in 30%–80% of male infertility cases [12]. Inflammation and OS are best understood as bidirectional partners rather than a strictly one‐way cascade. Cytokines and immune cells enhance ROS production, and ROS in turn activates innate immune responses through TLRs and inflammasomes, while oxidative posttranslational modifications create neoantigens that drive further immune activation [13]. Notably, oxidative stress can also be the initiating event: mitochondrial ROS and oxidized mitochondrial DNA are established triggers of NLRP3 inflammasome assembly, so that conditions in which oxidative stress arises first—such as varicocele, aging, metabolic disease, and primary mitochondrial dysfunction—can themselves ignite a secondary inflammatory response [14, 15, 16]. Regardless of the initiating direction, once established this inflammation–oxidative stress loop becomes self‐reinforcing, and ROS generated by both infiltrating leukocytes and spermatozoa converge on the same targets, promoting motility defects and DNA fragmentation [17, 18]. This bidirectionality helps explain the inconsistent results of antioxidant monotherapy: in men in whom oxidative stress is largely secondary to ongoing immune activation, targeting ROS alone does not remove the upstream inflammatory driver, whereas antioxidant therapy may still be an effective, mechanism‐appropriate option when oxidative stress is the primary or dominant pathway [19].

That said, not all inflammation is “bad” in the reproductive context [20]. The system depends on a physiological baseline level of inflammation. Thus, testicular and accessory gland cells routinely produce interleukin‐1 (IL‐1), interleukin‐6 (IL‐6), IL‐10, tumor necrosis factor‐alpha (TNF‐α), granulocyte colony‐stimulating factor (G‐CSF), and TGF‐β. These factors modulate germ cell proliferation, Sertoli‐germ cell interactions, steroidogenesis, and the immunoregulatory signature of seminal plasma [13]. Seminal TGF‐β, for instance, helps to establish immune tolerance of the female tract following ejaculation. Problems only arise when this homeostasis is disrupted. Some examples of this situation include when cytokine concentrations are inappropriately high or low, pro‐ and anti‐inflammatory signals are mismatched, or cytokine sources change due to infections, obesity‐related inflammation, chronic conditions, or even cancer therapies [13, 21]. In this instance, cytokines that underpin fertility instead disturb BTB integrity, initiate germ cell apoptosis, activate inflammasomes, disrupt epididymal development, and overwhelm antioxidant mechanisms. It is worth noting that infertile men tend to have a distinctive seminal cytokine profile, characterized by elevated levels of IFN‐γ, TNF‐α, IL‐12, and IL‐18, and low levels of regulatory cytokines. This has been reported to correlate with poorer semen quality and, in preliminary studies, with less favorable ART outcomes [22].

However, despite such obvious connections, the application of concepts related to inflammation to the clinical management of male infertility remains limited. The dominant paradigm has been ROS‐centered: quantify oxidative stress, supplement with antioxidants, and consider semen defects largely the result of redox imbalance. However, ROS alone does not explain many immune‐specific mechanisms now being recognized. These include antigen exposure leading to antisperm‐antibody development, injury mediated by complement and immune complexes, toll‐like receptor signaling in sperm and supporting structures, inflammasome activation in Sertoli and Leydig cells, and changes in the complex cytokine network [4, 23]. Each of these mechanisms may trigger a ROS response but can also cause injury through other mechanisms, including sperm agglutination, complement lysis, and irreversible fibrosis. None of which are fully reversible by antioxidants. Recent reports further extend this perspective. Key immune pathways involved include seminal proteomic changes, viral infections such as the Zika virus, which result in testicular inflammation, and rare genetic disorders, such as AIRE deficiency, that impair central immune tolerance [7, 11]. These findings in total underscore inflammation as a primary driver rather than an incidental consequence of male reproductive failure.

Implementing these insights in clinical practice requires an expanded diagnostic and therapeutic paradigm. Conventional semen analysis offers limited information regarding subclinical inflammation. We must standardize immune‐focused tools, including seminal cytokine profiling, leukocyte and neutrophil characterization, immune complex assessment, and advanced OS analyses [24]. In addition, the underlying driver of oxidative stress should first be identified, since this driver—which may or may not be inflammatory—determines whether antioxidants alone are likely to suffice. When inflammation is the identified driver, therapeutics may need to extend beyond simple antioxidants to include specific immunomodulatory approaches, such as cytokine‐targeting agents, complement inhibitors, locally delivered anti‐inflammatory therapies, and tolerance‐inducing strategies. The most significant benefits are likely to result from interventions that can address both the immune and oxidative components. One promising area is the study of how inflammation and OS affect germ cell epigenetics and the potential effects on the health of subsequent generations.

This review aims at merging immune elements—autoantibodies, inflammasomes, immune complexes, and cytokine imbalance—with those of oxidative processes to reconceptualize male infertility and foster progress toward improvement of outcomes.

## 2 Methods

The research followed a narrative synthesis approach, integrating systematic elements to achieve transparent, reproducible results across database searches, study screening, and interpretation. A structured database search across PubMed, Scopus, and Web of Science was executed to retrieve relevant studies from 2015 to 2025. The search protocol used Medical Subject Headings (MeSH) and specific keywords about inflammation and male reproductive health, including “inflammation AND male infertility,” “cytokines AND spermatogenesis,” and “oxidative stress AND sperm function,” and their equivalent terms. The core emphasis was on English‐language studies, and the search returned 500 records. Finally, the researchers reviewed the records for relevance using title and abstract screening.

The research group examined articles on how inflammation affects male reproductive health. Human clinical studies using observational, case–control, cohort, and interventional designs were prioritized because of their direct application in clinical practice. The accepted studies were conducted in vitro and in animals and provided valuable information on cytokine signaling, oxidative stress pathways, and sperm epigenetic regulation. The review excluded research on inflammatory diseases outside reproductive health, non‐fertility‐related conditions, review articles without original data, conference abstracts, and papers with insufficient methodological information.

The team's work aimed to conduct a full‐text evaluation of eligible studies to obtain information on research design, participant demographics, inflammatory marker data, sperm measurement results, experimental findings, and treatment outcomes. The review used a qualitative synthesis because studies used diverse methods and measured different outcomes with different biomarkers. The review analyzed elevated seminal TNF‐α and IL‐6 levels and leukocyte counts in infertile patients using existing meta‐analysis findings to strengthen the evidence.

The review did not conduct a PRISMA‐based risk‐of‐bias assessment because it fell short of the standards for a complete systematic review. The review team assessed study quality through indirect measures, including research participant numbers, experimental design, control group definition, and biomarker assessment methods. The review synthesis placed greater emphasis on studies that demonstrated high quality and substantial impact. The review maintains complete transparency by providing detailed descriptions of the search methods, inclusion criteria, and study selection process.

### 2.1 Inflammation in the Male Reproductive System

#### 2.1.1 Cellular and Molecular Mechanisms

Inflammatory‐induced infertility is a multifactorial mechanism. Infiltration of leukocytes into the seminal plasma, particularly macrophages and neutrophils, has a destructive role in the male genital tract [13]. Under physiological conditions, resident testicular and epididymal macrophages have an immunosuppressive M2 phenotype and secrete anti‐inflammatory cytokines such as interleukin‐10 (IL‐10) and transforming growth factor‐β (TGF‐β). In pathological states, these leukocytes differentiate into a pro‐inflammatory M1 phenotype. This leads to a significant increase in the production of pro‐inflammatory cytokines, including interleukin‐1β (IL‐1β), IL‐6, and TNF‐α [10].

In the acute lipopolysaccharide‐induced orchitis model, NLRP3 inflammasome activation and CCL2‐mediated recruitment result in neutrophil infiltration into testicular tissue. Thus, CD45^+^Ly6G^+^ cells increase to about 4–5 times the original level within 24 h. Neutrophil infiltration within the testis is primarily observed in this acute experimental model, whereas both macrophages and neutrophils are identified as the dominant leukocyte populations in seminal plasma during inflammation [14]. Leukocytospermia, defined as the presence of more than 1 × 10^6^ peroxidase‐positive leukocytes per milliliter of ejaculate, serves as the World Health Organization diagnostic threshold for genital tract inflammation [25]. Pro‐inflammatory cytokines and chemokines, notably IL‐1β, IL‐6, TNF‐α, and CXCL8 (also known as IL‐8), are key mediators of inflammatory damage [25]. Patients with genital tract inflammation have elevated levels of these mediators in seminal plasma, with proposed diagnostic thresholds of IL‐6 exceeding 30 pg/mL and IL‐8 exceeding 7000 pg/mL indicating active inflammation. These cytokines have direct damaging effects on sperm function and structure [22]. To be specific, TNF‐α, IL‐6, and IL‐1β suppress the expression of tight‐junction proteins, such as claudin‐11, and adherens junction proteins, including E‐cadherin and α‐catenin, between adjacent Sertoli cells [26]. The blood‐testis barrier, which is an important component for testicular immunity, becomes more permeable as a result of these changes. Permeability of the blood‐testis barrier leads to further leukocyte infiltration and to the exposure of previously sequestered postmeiotic sperm antigens to the systemic immune system. As a result, the inflammatory response shifts from an initial natural phase to an adaptive immunity response [17]. TNF‐α and TNF‐related apoptosis‐inducing ligand (TRAIL) are the primary mechanisms by which the inflammatory cascade induces sperm apoptosis. These extrinsic apoptotic signals lead to germ cell loss and compromised spermatogenesis [27]. Infiltrating leukocytes, especially neutrophils and activated macrophages, generate significant quantities of reactive oxygen species (ROS). In spermatozoa, excessive ROS can result in mitochondrial dysfunction, DNA fragmentation, and lipid peroxidation [28]. ROS overproduction is widely implicated as the principal mechanism of oxidative damage [17].

Damage to the blood‐testis barrier leads to the production of antisperm antibodies (ASAs) during autoimmune responses [4, 29]. Infertile men have higher levels of ASAs than fertile individuals. ASAs are significantly more common in conditions like varicocele, genital tract infection, trauma, vasectomy, and surgical procedures that are linked to an immune system defect [4]. IgG and especially secretory IgA antibodies are produced when the humoral immune system encounters sperm antigens. These antibodies bind directly to the sperm surface and impair fertility through multiple mechanisms: agglutinating ASAs induce sperm clumping and markedly reduce progressive motility, while immobilizing antibodies, acting through complement activation, completely halt motility [4]. Both antibody classes also negatively affect sperm penetration, zona pellucida binding, and the acrosome reaction. ASAs worsen oxidative stress and contribute to sperm DNA fragmentation [17, 26].

Inflammasomes cause testicular inflammation by NLRP3 activation [14, 16]. Lipopolysaccharide activates NLRP3 in testicular somatic cells, Leydig cells, and Sertoli cells. NLRP3 activation is essential for IL‐1β secretion, CCL2 production, and subsequent neutrophil recruitment in experimental orchitis models [14]. Increased seminal NLRP3 protein reported in varicocele patients due to upregulation of NLRP3 and subsequent IL‐1β and IL‐18 release has also been observed in primary Sertoli cells exposed to combined 2,3,7,8‐tetrachlorodibenzo‐p‐dioxin and lipopolysaccharide via the Klotho/PDLIM2/NF‐κB pathway. Mitochondrial dysfunction and ATP depletion result from TNF‐α and ROS‐mediated injury to spermatozoa and somatic cells [15, 30].

#### 2.1.2 Immune‐Mediated Sperm Damage

**FIGURE 1:**
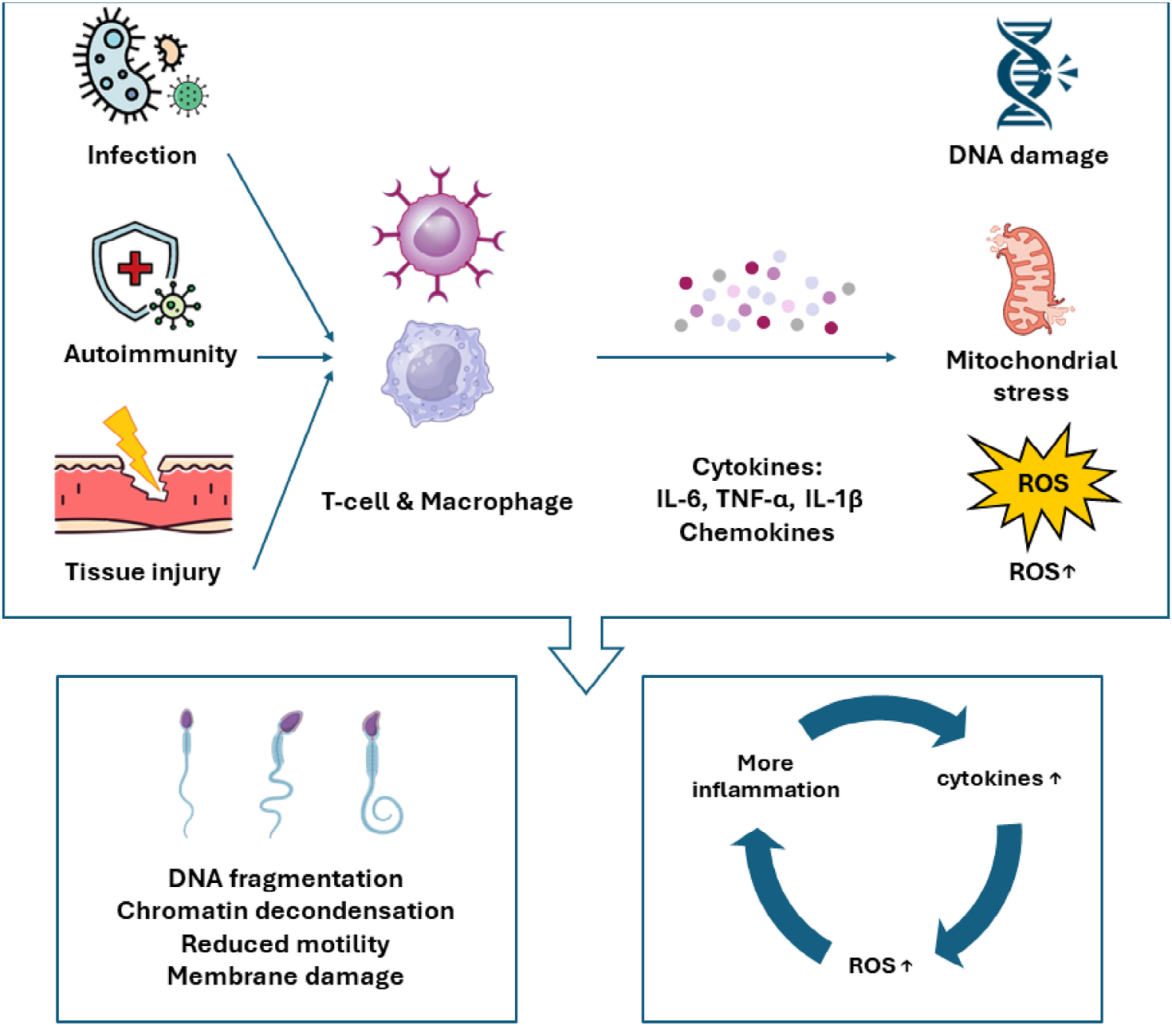
Overview of inflammation‐related pathways leading to sperm dysfunction.

Inflammation harms sperm through oxidative mechanisms that negatively affect genomic content. Sperm DNA fragmentation is frequently seen in inflammatory conditions, including varicocele, diabetes mellitus, and leukocytospermia [31, 32, 33]. Elevated levels of pro‐inflammatory cytokines and ROS produced by leukocytes are also observed [18]. Increased sperm DNA fragmentation is associated with reduced sperm motility and count. ROS plays a significant role in strand breaks [18, 28]. The second factor is ASAs that bind apoptosis‐related proteins such as caspase‐3 and heat shock proteins (HSP 70 and HSP 90) [29]. ASA‐positive infertile patients have higher levels of ROS, particularly mitochondrial ROS [29]. Overall, ROS and ASAs promote apoptotic DNA degradation. The molecular mechanisms of mitochondrial damage, which negatively affect sperm motility and viability, have not been fully identified [31]. Mitochondrial superoxide production is elevated in inflammatory states, such as those seen in type 2 diabetes mellitus [32]. These superoxides lead to increased lipid peroxidation, reduced mitochondrial membrane potential, and higher rates of sperm DNA fragmentation [32]. Pro‐inflammatory cytokines, particularly TNF‐α, have been reported to change mitochondrial function, increasing nitric oxide production. Therefore, malondialdehyde accumulates, and the acrosome reaction is inhibited (Figure 1) [31].

### 2.2 Factors Contributing to Inflammation‐Induced Infertility

Inflammation plays a critical role in the pathogenesis of male infertility. Both intrinsic and extrinsic factors contribute to chronic low‐grade or clear inflammatory states that affect the testicular immune microenvironment, spermatogenesis, and sperm function.

### 2.3 Intrinsic Factors

#### 2.3.1 Prostatitis and Male Accessory Gland Infections (MAGI)

Prostatitis and male accessory gland infections (MAGI), among intrinsic factors, represent a substantial share of inflammation‐induced infertility cases [34]. Chronic prostatitis/chronic pelvic pain syndrome (CP/CPPS, NIH Category III) and chronic bacterial prostatitis (Category II) are all associated with remarkable changes in semen parameters. Some of these changes are reduced sperm concentration, progressive motility, normal morphology, and increased DNA fragmentation [27, 35]. Elevated pro‐inflammatory cytokines in seminal plasma are associated with these changes. Multiple studies report increased levels of IL‐6, TNF‐α, IL‐1β, IL‐8, and IFN‐γ. IL‐6 and TNF‐α directly disrupt mitochondrial respiration in spermatozoa, increase mitochondrial ROS production, damage mitophagy, and trigger an energy crisis. Thus, apoptosis and DNA damage experience an increase [34, 35].

#### 2.3.2 *Chlamydia trachomatis*

*Chlamydia trachomatis* ( *C. trachomatis* ) is the most common bacterial sexually transmitted infection worldwide. Men often appear asymptomatic, and the bacteria can persist for years in their genital tract. The most affected organs by the infection are the urethra, epididymis, or prostate. Epididymitis, chronic bacterial prostatitis, and MAGI are the significant problems caused by *C. trachomatis* . This bacterium induces inflammation via lipopolysaccharides, leading to leukocytospermia and elevated cytokines [8, 36].

#### 2.3.3 Varicocele

Varicocele is present in 35%–40% of infertile men [26, 37]. This condition is consistently linked to oxidative stress, ROS overproduction, lipid peroxidation, and a chronic inflammatory state [33, 37]. Hypoxia and venous stasis are major pathophysiological factors of hypoperfusion that lead to nutrient deprivation and testicular immune dysregulation. The blood‐testis barrier is also harmed by downregulation of claudin‐11, E‐cadherin, and α‐catenin [33, 37]. Elevated seminal and tissue levels of IL‐1β, IL‐6, IL‐8, IL‐18, TNF‐α, and IFN‐γ are reported in both human varicocele patients and experimental models [26, 33]. NLRP3 inflammasome activation in testicular macrophages represents a recurrent mechanism driving IL‐1β maturation and testosterone inhibition [26].

### 2.4 Extrinsic Factors

#### 2.4.1 Environmental Toxicants

Lifestyle‐associated molecular patterns (LAMPs) and environmental toxicants are potential activators of NLRP3 [16]. Bisphenol A (BPA), widely used in plastic manufacturing, is a pollutant that disrupts the endocrine system [38]. BPA binds to estrogen receptors in testicular cells, inducing a Th1/Th2 imbalance [39]. Pro‐inflammatory Th1 responses increase and suppress regulatory immunity. Testicular inflammation and oxidative stress induced by BPA decrease sperm quality [38, 39]. A study by Yang et al., summarized in a recent review, reported that BPA exposure in male mice led to testicular ferroptosis [40]. Ferroptosis is accompanied by less expression of GPX4 and FTH, and an increase in COX2 and ACSL4, mitochondrial damage, and iron accumulation [40].

Di‐(2‐ethylhexyl) phthalate (DEHP) causes inflammation in the male reproductive system in multiple ways. Prenatal DEHP exposure at a specific dose (300–750 mg/kg) in rats significantly reduced anogenital distance, sperm count, and motility [41]. It also increased testicular oxidative stress and pro‐inflammatory cytokines, such as IL‐6 and TNF‐α [41, 42]. Simultaneously, DEHP suppresses Th1‐related IFN‐γ and enhances Th2 cytokines such as IL‐4 and IL‐13. Germ cell apoptosis and Leydig cell dysfunction are associated with a Th2‐biased environment [41, 42]. In adult mice, chronic low‐dose DEHP (40 μg/kg/day) damages the blood‐testis barrier. This alteration downregulates occludin and claudin‐11 (tight‐junction proteins) and activates NF‐κB signaling in Sertoli cells. As a result, macrophages infiltrate, and testicular inflammation is sustained [43]. Men in the highest quartile of urinary MEHP (mono‐2‐ethylhexyl phthalate, the primary DEHP metabolite) exhibit 34%–42% lower total sperm count and progressive motility, alongside elevated seminal plasma IL‐6 and reduced IFN‐γ, indicating a pro‐inflammatory profile [44].

Lead (Pb) exposure promotes a Th2 response and has been associated with inflammation‐related male infertility. Semen analysis of workers occupationally exposed to lead, with blood lead levels > 30 μg/dL, reveals oxidative damage consistent with marked oligospermia and asthenospermia [45]. Workers with high lead exposure have also been reported to show altered seminal cytokine profiles, with higher IL‐4 and lower IFN‐γ. Lead induces mitochondrial ROS generation in Leydig and Sertoli cells, which then activates the NLRP3 inflammasome and caspase‐1‐dependent IL‐1β release. Thus, macrophage‐driven testicular inflammation occurs. This inflammation interferes with the expression of steroidogenic acute regulatory (StAR) protein and testosterone synthesis. Eventually, lead damages the blood‐testis barrier, promotes the formation of antisperm antibodies, and perpetuates Th2‐biased immune responses [45].

#### 2.4.2 Obesity

Obesity is a crucial extrinsic pro‐inflammatory factor. High BMI is associated with poor sperm parameters [46]. Animal models demonstrate that suppressed hypothalamic–pituitary–gonadal axis function reduces testosterone and semen quality [21]. The obesity–inflammation–aromatase axis causes adipose tissue hypertrophy, which increases pro‐inflammatory cytokines (IL‐6, TNF‐α, IL‐1β, IL‐18) [21]. In addition, adipocyte CYP19 (aromatase) is regulated by PPARγ, leading to excessive androgen‐to‐estrogen conversion, hormonal imbalance, and impaired spermatogenesis. In meta‐inflammation, adipokines are dysregulated with leptin increasing and adiponectin decreasing. Macrophages are also polarized to an M1 phenotype. These macrophages induce oxidative stress in testicular tissue, leading to complications such as hypogonadism and reduced sperm quality [21].

#### 2.4.3 Age and Autoimmunity

Aging, especially paternal age exceeding 40 years, increases the risk of autoimmune responses by elevating ASA levels and NLRP3 expression [16, 47]. The relationship between inflammation and aging is described as “inflammaging.” This state impacts all tissues, including the male reproductive system. Aging is associated with increased testicular inflammation, higher macrophage numbers, ultrastructural changes, and elevated pro‐inflammatory cytokines (e.g., IL‐1β, IL‐6, TNFα) [48, 49]. Inflammaging correlates with reduced androgen production (e.g., testosterone), decreased testicular size, fewer Sertoli and Leydig cells, and altered spermatogenesis (e.g., accelerated spermatocyte apoptosis) [48, 49]. Macrophages hyperactivate with age, increasing COX2 and prostaglandins (inflammatory mediators), which then suppress testosterone production [48]. As a result, a decline in steroidogenesis is observed due to increased ROS and lipid peroxidation in Leydig cells, caused by oxidative stress [48].

Studies in GH‐transgenic mice with accelerated aging show elevated levels of COX2, prostaglandins, macrophages, lipid peroxidation, and apoptosis. These animal articles describe the effect of aging on male infertility [49].

Variations in the human leukocyte antigen (HLA) system regulate immune responses. HLA Class I and II alleles, such as HLA‐B27 and HLA‐DR, are associated with a higher risk of chronic inflammatory diseases [50]. Oligoasthenoteratozoospermia often leads to chronic genital inflammatory responses. Elevated TNF‐α and IL‐17 levels compromise the blood‐testis barrier (BTB) and cause germ cell apoptosis. This genetic interaction causes chronic epididymitis and leukocytospermia by presenting altered self‐peptides to autoreactive CD4+ T cells, which promote a Th1‐dominant response that damages Sertoli cells and compromises the acrosome reaction [51]. HLA‐G polymorphisms, which affect immune tolerance in the testis, are linked to idiopathic infertility [52]. A 2022 genome‐wide association study found that HLA‐G*01:04 increases TNF‐α/IL‐10 ratios by 1.8‐fold, is associated with increased oxidative stress, and reduces sperm motility [53]. This inflammation leads to elevated plasma IFN‐γ and harmed sperm capacitation [51].

#### 2.4.4 Psychological Factors

Mental stress, chronic anxiety, and depression are contributing factors to male infertility by promoting systemic inflammation and oxidative stress. Long‐term psychological stress activates the hypothalamic–pituitary–adrenal (HPA) axis, elevating glucocorticoid levels [54]. If glucocorticoid receptors function in a poorly regulated manner, the MAPK and NF‐κB pathways are activated in testicular cells, leading to increased reactive oxygen species and pro‐inflammatory cytokines [55, 56]. This inflammatory phase suppresses spermatogenesis, reduces sperm motility and viability, and increases DNA fragmentation, linking stress with subfertility [55, 56]. Research shows that men with depression have higher levels of seminal oxidative stress markers, such as malondialdehyde (MDA). These markers correlate with asthenozoospermia and teratozoospermia. Psychological distress has been reported to dysregulate the PDK‐PDC mitochondrial axis and ATP production, and defective ATP production weakens sperm motility [57, 58, 59] (Table 1).

**TABLE 1:** Key factors and their immune contributions.

Factor category	Specific examples	Immune mechanisms	Evidence from studies	Impact on sperm	References
Intrinsic: Infections	*Chlamydia trachomatis* , *Neisseria gonorrhoeae*	IL‐6, TNF‐α upregulation, leukocyte infiltration	CDC STI Treatment Guidelines (2021), RCTs	Reduced motility, DNA fragmentation	[60, 61]
Intrinsic: Autoimmune diseases	Rheumatoid arthritis, inflammatory arthritis	TNF‐α, IL‐1β cytokine elevation, systemic inflammation	Perez‐Garcia et al. (2022)	Increased sperm DNA damage, poor quality	[62]
Intrinsic: Leukocytospermia	Elevated seminal WBCs (> 1 × 10^6^/mL)	ROS production, cytokine secretion (IL‐6, TNF‐α)	WHO manual, clinical observational studies	Oxidative DNA damage, reduced motility	[63]
Extrinsic: Environmental pollutants	Bisphenol A (BPA), heavy metals	Oxidative stress, disruption of cytokine signaling	Reviews on environmental impact, animal models	Lower sperm count, motility defects	[64, 65]
Intrinsic: Varicocele	Varicocele‐induced testicular inflammation	Cytokines IL‐6, ROS from immune cells	Clinical trials on anti‐inflammatories + surgery	Impaired spermatogenesis, increased ROS	[66, 67]
Extrinsic: Diet and lifestyle	Western diets, smoking	Elevated systemic IL‐6, oxidative stress	Nutrition epidemiology (Mediterranean diet impact)	Decreased concentration and motility	[68, 69]
Intrinsic: Inflammation‐triggered epigenetic changes	Aberrant DNA methylation, histone modification	Methylation defects altering imprint genes	Epigenetic studies linking paternal inflammation to autism risk	Offspring risks, impaired sperm function	[70, 71]
Intrinsic: Oxidative stress	Excess ROS from immune response	Lipid peroxidation, DNA strand breaks	Sperm oxidative stress studies	DNA fragmentation, telomere shortening	[66]

### 2.5 DNA Damage, Repair, and Epigenetic Consequences

The structural cohesion of DNA in spermatozoa is critical for the proper passage of genetic information to offspring and for the facilitation of healthy embryogenesis [72]. However, the DNA in spermatozoa is more vulnerable to inflammation‐driven damage than that of somatic cells [73]. There is evidence of increased DNA vulnerability in spermatozoa due to inflammation, which can create an environment beyond the spermatozoa's repair mechanisms in the male genital tract. This leads to fixed genetic defects with broad‐ranging consequences for gene expression [74].

Relatively speaking, repair mechanisms available to a sperm cell's genome are much more restricted than those of somatic cells. These cells primarily rely on base repair mechanisms, with OGG1 as a key enzyme for repairing oxidative damage. On the other hand, nucleotide excision repair, a mechanism particularly efficient in dealing with bulky adducts and helix‐distorting damage, is entirely lacking in mature spermatozoa [75]. This minimal repair machinery means that once the sperm cell's genome is damaged by oxidative stress or single‐ or double‐strand breaks, the likelihood of repair decreases significantly [28]. Inflammation increases these risks by enhancing ROS production and inflammatory cytokine synthesis, leading to strand breaks and base damage [73]. Because of the constant repair of the DNA, the adverse effects of the damage induced in the sperm would be seen later on in the offspring's life [76]. Telomere breakdown due to inflammation would be a crucial factor here, as the cytotoxic effects of inflammation increase telomere shortening, leading to cellular aging and death [77]. It is relevant to acknowledge that telomeres play a pivotal role in mitigating the cytotoxic effects of inflammation by protecting the chromosomal ends from destruction induced by the inflammatory response triggered by the genetic defect in the cells [78].

Furthermore, inflammation‐induced DNA damage leads to the establishment of specific mutational spectra, including GC‐to‐TA transversions, which represent base substitutions in which guanine‐cytosine base pairs are replaced with thymine and adenine base pairs. This is due to the presence of products of oxidative damage, including 8‐Oxoguanine, which form base pairs instead of guanine [79]. The presence of these specific mutation points within the spermatogenic cell's DNA would be detrimental to gene function because they can interfere with normal transcriptional processes, alter protein structure, and ultimately suppress embryonic development [80]. In addition to direct genetic toxicity, inflammation alters epigenetic marks in sperm, particularly through DNA methylation. The methylation of sperm DNA is fundamental in defining the imprinting and gene expression requirements for embryonic development [81]. Oxidative stress induced by inflammation after cytokine exposure may disrupt the activity of enzymes involved in hypomethylation or hypermethylation [82]. Alterations in paternal expression disrupt the expression of the imprinted gene. The transferred epigenetic changes caused by paternal inflammation can be passed on to the next generation, influencing offspring phenotype without altering the gene sequences [83]. There is evidence from epidemiological studies relating paternal inflammatory conditions to an elevated vulnerability of offspring developing neurodevelopmental disorders, such as autism spectrum disorder (ASD), among other diseases [84]. Thus, inflammation is a destructive factor for sperm DNA integrity because it overpowers an already precarious ability to repair DNA, leading to an immense amount of repair‐resistant damage, including telomere loss and the buildup of mutations. Simultaneously, inflammation‐induced epigenetic changes, including methylation and imprinting, further increase the already elevated risks of unanticipated outcomes in offspring.

### 2.6 Clinical Implications and Transgenerational Effects

Inflammation of the male reproductive tract, when present in the father, manifests as specific infertility syndromes with profound implications for pregnancy outcomes and offspring health. The evidence on the impact of paternal health on conception rates has catalyzed numerous studies aimed at understanding inflammation‐induced mechanisms underlying declining paternal fertility and intergenerational paternity risks [85].

### 2.7 Infertility Phenotypes

Inflammation of the male genital tract is manifest by specific semen characteristics, most notably asthenozoospermia and teratozoospermia [74]. Specific pro‐inflammatory cytokines, such as TNF‐α, IL‐1β, IL‐6, and IFN‐γ, directly affect sperm function. Cytokines directly induce ROS generation, a process known as oxidative stress, which promotes apoptosis in germ cells and disrupts the blood‐testis barrier [51]. Leukocytospermia, an increased seminal fluid leukocyte count, is a defining criterion of inflammation of the genital tract and is an indicator of exaggerated immune system activation associated with adverse effects on sperm quality [86]. However, its predictive value for male infertility remains debated, as several studies have found it to correlate poorly with conventional semen parameters and clinical outcomes [87]; it is therefore best interpreted alongside, rather than in place of, direct markers of inflammation. Migration of immune cells, including macrophages, T cells, and mast cells, into the testes and epididymis promotes inflammation via cytokine secretion and the production of reactive oxygen species. It is also a factor in hypogonadism due to inflammation of the Leydig cells, which negatively impacts spermatogenesis, sperm maturation, and overall sperm function [88].

### 2.8 Pregnancy Outcomes

Paternal inflammatory conditions have a substantial impact on pregnancy outcomes, including an increased risk of recurrent pregnancy loss and failure of assisted reproduction therapy [62, 89, 90]. There is evidence suggesting an increased miscarriage risk among partners of men with inflammatory arthritis, independent of the established risks of parental age, among other factors [62]. A theoretical model suggests that the underlying cause may be systemic inflammation's effect on sperm DNA, leading to aberrations in DNA function and preventing embryonic growth [91]. Reduced sperm quality because of inflammation translates to low rates of fertilization and low‐quality embryos in the ART setting. Intracytoplasmic sperm injection using spermatozoa retrieved from men with genital tract inflammation is linked with an increased miscarriage rate because of unrepairable DNA defects passed to the embryos. This inflammation is also known to interfere with important immune interactions between the male and female reproductive systems, a condition vital for implantation and placentation [64].

### 2.9 Offspring Burden

**FIGURE 2:**
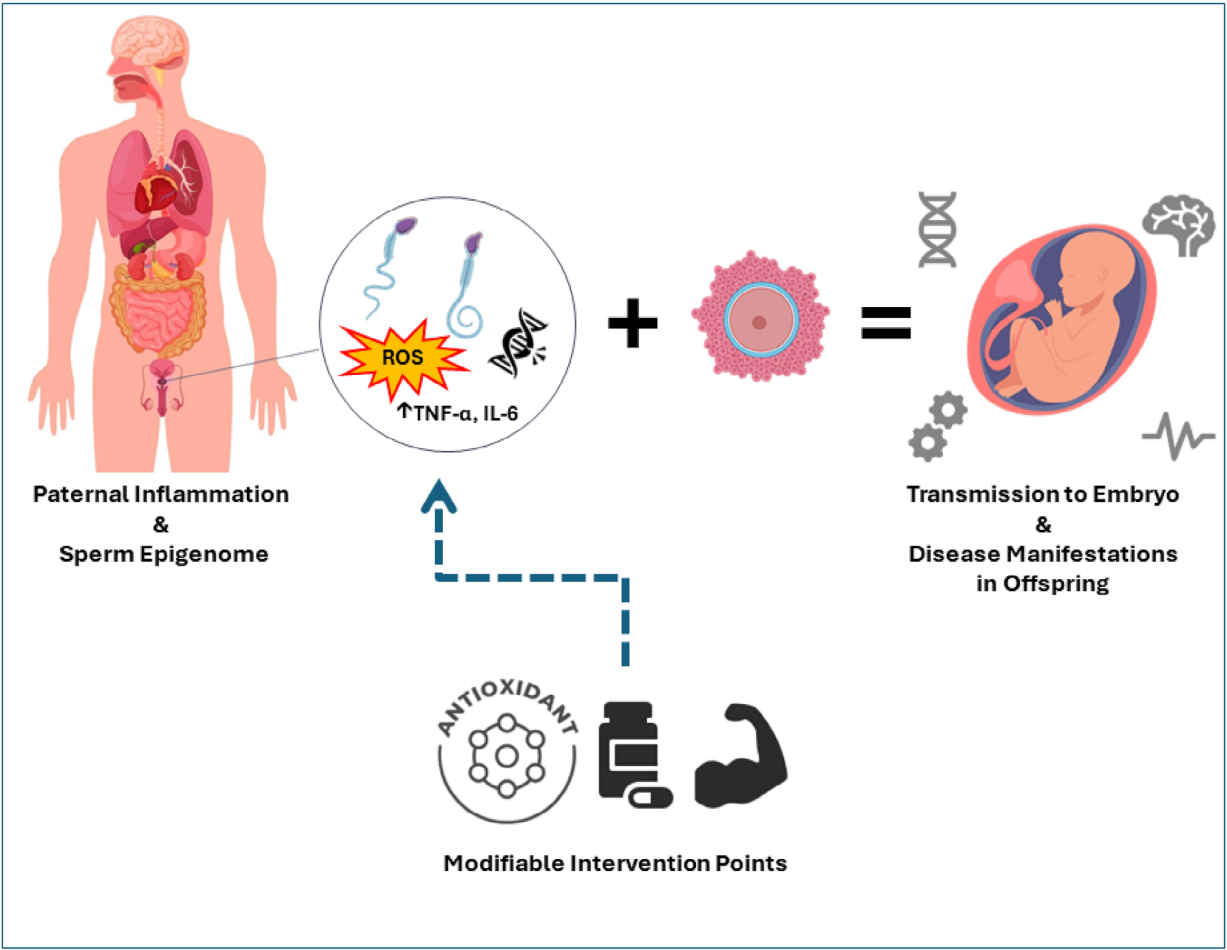
Schematic representation of clinical and transgenerational pathways linking paternal inflammation to offspring health.

The intergenerational effects of paternal inflammation include those associated with pregnancy complications, but reach further to include lifetime risks of offspring morbidity [92]. This is because inflammation‐driven changes in sperm DNA methylation and other inflammatory effects on sperm RNA content can be transferred to offspring and increase vulnerability to disorders such as neurodevelopmental disorders and cancers. Sperm RNA cargo, including microRNAs and other small RNAs, is known to be involved in the regulation of early embryonic development and gene expression. However, inflammatory disease alters this RNA transcriptome, potentially leading to dysregulation of developmental programming [93]. The resulting epigenetic dysregulation from paternal inflammation leaves a molecular stamp that persists beyond conception and affects the offspring's phenotype and disease vulnerability through mechanisms of nongenetic inheritance. The passage of molecules from one generation to the next underscores the importance of targeting male reproductive tract inflammation in preconception optimization therapies. In this way, they can be used to alleviate both the acute effects of fertility and the risks to offspring (Figure 2, Table 2).

**TABLE 2:** Diagnostic tests and biomarkers for inflammation‐related damage.

Test/biomarker	Sensitivity/specificity (approx.)	Clinical threshold (example)	Pros/cons	Relevance to mechanisms	References
TUNEL assay (sperm DNA fragmentation)	Sensitivity: 96.6% Specificity: 87.5%	DFI > 20.3% indicates high DNA damage	Pros: Direct measure of strand breaks; widely used. Cons: Requires flow cytometry or fluorescence microscopy.	Detects DNA strand breaks consistent with cytokine/ROS‐driven damage and failed repair (limited BER, absent NER); not a specific diagnostic test for inflammation, and a normal result does not exclude subclinical inflammation.	[94, 95]
SCSA (sperm chromatin structure assay)	Sensitivity: 75%–79% Specificity: 85%–86%	DFI > 25%–30% associated with RPL/ART failure	Pros: High throughput, standardized; good prognostic data for ART. Cons: Requires specialized equipment.	Reflects chromatin instability from oxidative stress, telomere attrition, and GC:TA transversions; like TUNEL, a general marker of DNA/chromatin damage rather than a test specific to inflammation.	[95, 96]
Seminal IL‐6 concentration	Sensitivity: 60%–75%; specificity: 60%–80%	IL‐6 > 50 pg/mL suggests active inflammation	Pros: Direct inflammatory cytokine marker; links to genital tract inflammation. Cons: Overlap with systemic inflammation; assay cost.	Indicates local cytokine milieu driving ROS production and overwhelming sperm repair capacity.	[97]
Seminal ROS/ORP (oxidation–reduction potential)	Sensitivity: 70%–90%; specificity: 60%–80%	ROS above lab upper limit or ORP > 1.4 mV/10^6^ sperm	Pros: Functional readout of oxidative stress; relatively quick. Cons: Pre‐analytical variability; not etiology specific.	Quantifies oxidative burden causing base oxidation, strand breaks, and telomere erosion.	[66]
Leukocytospermia (peroxidase‐positive WBC)	Sensitivity: 60%–70%; specificity: 70%–80%	> 1 × 10^6^ WBC/mL semen	Pros: Simple, inexpensive; WHO‐recognized marker. Cons: Indirect; misses sterile inflammation.	Reflects immune‐cell influx producing ROS and cytokines that damage sperm DNA and epigenetic marks; predictive value for infertility outcomes is debated, and it misses sterile (non‐leukocytic) inflammatory processes.	[51]
Seminal TNF‐α/IL‐1β	Sensitivity: 50%–70%; specificity: 60%–80%	Above assay‐specific upper normal range	Pros: Pathway‐specific inflammatory markers. Cons: Not routinely available; variable cutoffs.	Mirror NF‐κB–driven inflammatory signaling that promotes apoptosis and DNA damage.	[67, 74, 96]
γH2AX (DNA double‐strand breaks)	Emerging: 80%–90% sensitivity (preclinical)	> 5% positive sperm	Pros: Specific DSB marker. Cons: Research focused.	Reflects DNA double‐strand breaks that can result from cytokine/ROS exposure and failed repair; not specific to an inflammatory etiology	[64]
Epigenetic assays (sperm DNA methylation/imprinting panels)	Sensitivity/specificity still emerging; research use	No uniform clinical cut‐off; locus‐specific deviations from reference	Pros: Direct assessment of heritable epigenetic alterations. Cons: High cost; currently research focused.	May capture methylation/imprint changes associated with paternal inflammation and offspring risks (e.g., neurodevelopmental disorders, cancer), but such changes are not specific to inflammation and can arise from other exposures.	[70, 71]

### 2.10 Therapeutic and Preventive Strategies

The control of inflammation in the male genital tract requires a holistic approach that considers pharmaceutical treatment, immunotherapy, lifestyle changes, and novel therapies. There is a need for an evidence‐based treatment approach in order to mitigate the potential effects of inflammation on reproduction and prevent transgenerational effects.

### 2.11 Pharmacological Interventions

#### 2.11.1 Anti‐Inflammatory Agents

Nonsteroidal anti‐inflammatory drugs (NSAIDs) are one of the first‐line drugs for chronic inflammation of the male genital tract. Other medications, including ibuprofen, rofecoxib, and celecoxib, have shown beneficial effects in treating inflammation, especially that associated with varicocele and prostatitis [98]. NSAIDs work by inhibiting cyclooxygenases, thereby reducing prostaglandin production and, in turn, inflammation. However, one must always be aware of the potential complications of these drugs despite providing beneficial treatment, especially in relation to gastrointestinal and renal contraindications, among others.

#### 2.11.2 Biologic Therapy

Targeted biologic therapies directed against specific inflammatory mechanisms offer promising, though still investigational, alternatives for autoimmune‐associated inflammation of the genital tract [99]. Antitumor necrosis factor‐alpha therapies, including infliximab, have demonstrated effectiveness in those with autoimmune disease involving infertility, including those with inflammatory arthritis [99]. Biologic therapies function by blocking inflammatory cytokines, with the potential to harm spermatogenesis and sperm function directly [100]. Low‐dose corticosteroids and mast cell stabilizers serve other purposes in immune modulation in selective cases of inflammatory disease. However, these must be used cautiously due to both the risks of immune suppression and potential metabolic effects [100].

#### 2.11.3 Antimicrobial Therapy

Infectious causes precipitating inflammation of the reproductive tracts require antibiotic treatment. Decisions on antibiotic treatment regimens would depend on the identified isolates and their antibiotic sensitivities. For example, in acute bacterial epididymitis with a sexually transmitted infection, a combination of ceftriaxone and doxycycline is prescribed, per international guidelines [60]. In contrast, fluoroquinolones such as ofloxacin and levofloxacin are used for gram‐negative infections [60, 101]. The duration of antibiotic therapy is 2–4 weeks, depending on the severity of the infection. Given the risk of antimicrobial resistance and gut dysbiosis, culture‐guided therapy with confirmation of pathogen clearance is recommended, and treatment should focus on eliminating the causative bacteria while preventing the sequelae of chronic inflammation.

#### 2.11.4 Immunomodulatory and Nutritional Approaches

Omega‐3 PUFA has anti‐inflammatory properties, including the suppression of proinflammatory cytokines, such as interleukin‐6 (IL‐6) [66, 102]. Omega‐3 PUFA balances inflammatory pathways and increases antioxidant activity within seminal fluid. Omega‐3 PUFA supplements would be beneficial for enhancing sperm quality characteristics in infertile males with inflammation‐induced infertility [66, 102]. Probiotic supplements can be considered a new approach based on the gut‐testis axis, according to which the composition of the intestinal microbiota can modulate systemic and local immune responses within the reproductive organs [65, 103]. Beneficial bacteria can potentially regulate immunity and inflammation, although further work is needed to identify the most effective strains and doses. Micronutrient therapy with zinc, selenium, vitamin E, and coenzyme Q10 may enhance the antioxidant capacity of seminal fluid and help with inflammation‐induced oxidative stress. They may also reduce ROS levels and improve sperm motility in cases of asthenozoospermia [104, 105].

#### 2.11.5 Lifestyle Modifications

Practices such as yoga and meditation that focus on both mind and body have shown measurable anti‐inflammatory effects [68, 106]. According to randomized controlled trials, regular meditation for 3 months can decrease IL‐6 levels by 20% and other inflammatory mediators [68]. Stress management therapies impact the HPA axis and the SNS, leading to reduced synthesis of inflammatory mediators [107]. Diets can affect levels of inflammation in the body. For instance, a Mediterranean diet consisting of fruits, veggies, grains, olive oil, and fish is rich in anti‐inflammatory ingredients such as polyphenols, antioxidants, and omega‐3 fatty acids. A lifestyle involving anti‐inflammatory diets can reduce inflammation and improve semen quality, according to observational studies (Table 3) [69, 110].

**TABLE 3:** Therapeutic interventions and evidence.

Intervention type	Mechanisms	Clinical evidence	Outcomes/impacts	Limitations/side effects	References
Pharmacological: NSAIDs (ibuprofen, celecoxib)	COX inhibition → ↓ prostaglandins, ↓ cytokines	RCTs in prostatitis/varicocele show ↓ inflammation, ↑ motility	Reduced seminal ROS, improved sperm parameters	GI ulcers, renal impairment, CV risks	[98]
Pharmacological: Anti‐TNF biologics (infliximab)	TNF‐α neutralization → ↓ cytokine cascade	Improved IVF success in inflammatory arthritis/IBD	↓ Miscarriage rates, better embryo quality	Infection risk, high cost, IV administration	[99]
Antibiotics (ceftriaxone + doxycycline)	Eradicate bacterial pathogens → ↓ leukocytospermia	CDC guidelines: STD epididymitis resolution	Restored sperm quality postinfection	Resistance, gut dysbiosis, allergy risks	[61]
Antioxidants (zinc, selenium, vit E, CoQ10)	↑ Seminal TAC, ↓ ROS, protect DNA repair	RCTs: ↓ DFI 20%–30%, ↑ motility 12%–18%	Improved asthenozoospermia, better ART	GI upset, optimal dosing unclear	[104, 108]
Omega‐3 PUFA supplements	↓ IL‐6, ↑ antioxidants, membrane stabilization	RCT 238 men: ↑ sperm count/motility	Enhanced semen quality in OAT	Fishy aftertaste, bleeding risk at high dose	[66, 102]
Probiotics (gut‐testis axis)	Restore microbiota → ↓ systemic inflammation	Preclinical: ↓ testicular inflammation	Improved spermatogenesis (animal models)	Strain/dose optimization needed	[65, 103]
Lifestyle: Mediterranean diet	Polyphenols/ω‐3 → ↓ NF‐κB, ↓ IL‐6/CRP	Meta‐analysis 33 RCTs: ↓ inflammation markers	↑ Semen quality (observational)	Adherence challenges, long‐term commitment	[69]
Mind–body: Yoga/meditation	HPA/SNS modulation → ↓ IL‐6 (20%), ↓ cortisol	RCTs: ↓ IL‐6/TNF‐α after 8–12 weeks	Reduced oxidative stress markers	Time‐intensive, variable practitioner skill	[68, 106]
Emerging: NLRP3 inhibitors (MCC950)	Blocks IL‐1β/IL‐18 → ↓ inflammasome activation	Preclinical efficacy in inflammation models	Potential ↓ sperm DNA damage (theoretical)	Clinical trials ongoing, safety unknown	[109]
Low‐dose corticosteroids	NF‐κB transrepression → ↓ pro‐inflammatory cytokines	Selective immune modulation in autoimmune cases	↓ Inflammation in specific protocols	Immunosuppression, metabolic effects	[100]

### 2.12 Future Directions

New treatment targets arise from advances in research on inflammatory pathways. Inflammasome inhibitors, including the selective inhibitor of NLRP3 inflammasome activation, MCC950, look promising in clinical trials for inflammatory diseases and may be relevant in the field of male reproductive medicine [109, 111]. The selective inhibitors target important inflammatory complexes independently without causing broad immune suppression. Personalized immune profiling: A paradigm shift toward precision medicine is embodied in the use of “pre‐assisted reproductive technology immune profiling.” Evaluation of both inflammatory biomarker levels and seminal leukocyte numbers will enable targeted treatment based on the underlying pathophysiologic mechanisms. This approach offers the potential to optimize treatment efficacy and prevent unnecessary therapies and their consequent adverse effects, thereby enhancing outcomes and reducing risks to offspring health associated with paternal inflammation.

## 3 Discussion and Perspectives

This review clearly highlights the central role of inflammation in the pathophysiology of male infertility. While varicocele and environmental factors have often been the focus of attention [33], the present paper emphasizes genitourinary infections, particularly subclinical and chronic entities such as *Chlamydia trachomatis* infection and chronic prostatitis, as manageable and potentially treatable targets [36]. These infections trigger an inflammatory response that initiates a destructive cascade, ultimately leading to oxidative stress. Firstly, the pathogens themselves and the attendant leukocytic response directly elevate the production of reactive oxygen species (ROS). Secondly, pro‐inflammatory cytokines such as TNF‐α and IL‐6 impair mitochondrial function in spermatozoa and testicular somatic cells, thereby further promoting ROS leakage [34, 35]. This bidirectional link creates a broken cycle of “inflammation → oxidative stress → further inflammation,” and explains why antioxidant therapy alone, in the absence of control over the underlying infectious and inflammatory drivers, frequently fails.

To move beyond the substantial overlap with previous ROS‐centered reviews of male infertility, the mechanisms discussed above can usefully be classified into two categories. (1) ROS‐dependent mechanisms, in which inflammation acts principally by amplifying oxidative stress: leukocyte‐ and sperm‐derived ROS causing lipid peroxidation, mitochondrial dysfunction, and oxidative DNA strand breaks [17, 18, 28]. (2) ROS‐independent, immune‐mediated mechanisms, which damage sperm and impair fertility through pathways that do not require ROS as an intermediate: antisperm‐antibody‐mediated agglutination and immobilization, complement activation and immune‐complex deposition [4, 23], TNF‐α/TRAIL‐induced extrinsic (receptor‐mediated) apoptosis [27], cytokine‐driven suppression of Sertoli‐cell tight‐junction proteins and consequent blood‐testis barrier disruption [26], and HLA‐restricted autoreactive T cell responses [50, 51]. This ROS‐independent category has received comparatively little attention in conventional oxidative‐stress‐centered paradigms, and we propose it as one of the more distinctive contributions of an inflammation‐centered framework: these pathways are not expected to respond to antioxidant therapy at all, regardless of patient selection, and instead require immune‐directed diagnostics and treatment.

It should be emphasized that immunological management is intended to complement, not replace, the established pillars of andrological care (surgical correction of varicocele, treatment of endocrine abnormalities, lifestyle optimization, and assisted reproduction); transitioning toward incorporating this immunological paradigm faces significant challenges. The largest obstacle is the considerable interindividual heterogeneity in immune responses. As noted above, cytokine profiles are shaped by multiple factors, ranging from genetic predispositions (e.g., HLA polymorphisms) to lifestyle determinants [50, 112]. Such heterogeneity implies that a single intervention (for instance, a uniform anti‐inflammatory regimen) is unlikely to be effective for all patients. A second key limitation is the shortage of standardized, reliable, and cost‐effective biomarkers for precisely quantifying male genital tract inflammation. Although cytokines detectable in semen, such as IL‐6 and IL‐8, can be measured [22, 25], uncertainties about definitive cutoffs and cost limit their routine use. Likewise, although leukocytospermia is a simple surrogate marker, it is indirect and fails to capture sterile inflammatory processes (e.g., varicocele), which are increasingly recognized as clinically important [86].

## 4 Conclusions

Future efforts to overcome these barriers should be in two key lines of action. First, longitudinal cohorts of infertile men with serially collected semen, blood, and clinical data are essential; such rich datasets would provide a firm foundation for understanding the timing and dynamics of inflammation and its consequences. In addition, the application of artificial intelligence and machine learning to analyze these complex datasets offers great promise. AI algorithms can be trained to identify distinct cytokine‐profile signatures and “AI‐driven cytokine profiling” associated with different etiologies (infectious, autoimmune, environmental) and differential responses to interventions. This approach could enable immune‐based subtyping of male infertility and ultimately support management in which the therapeutic strategy (antibiotic, anti‐inflammatory, immunomodulatory, or lifestyle intervention) is selected according to an individual's unique immune‐inflammatory fingerprint.

Although the inflammatory pathophysiology of male infertility is complex, the present findings provide a hopeful outlook. Increasing understanding of the inflammation–oxidative stress axis, together with an expanding therapeutic armamentarium, from targeted anti‐infective and biologic therapies to nutritional and behavioral interventions, makes it realistic to reduce the burden of infertilities with an inflammatory component. The key to achieving this is a decisive shift away from one‐size‐fits‐all strategies toward a personalized framework in which treatment is guided by a precise assessment of each patient's immuno‐inflammatory status. Such a paradigm shift in infertility management would not only increase the likelihood of achieving pregnancy but also reduce the transmission of mutations and sperm‐borne epigenetic disturbances [80].

## Funding

The authors have nothing to report.

## Ethics Statement

The authors have nothing to report. This is a narrative review article based on previously published studies; no new experiments involving humans or animals were conducted.

## Conflicts of Interest

The authors declare no conflicts of interest.

## Data Availability

Data sharing is not applicable to this article as no datasets were generated or analyzed during this study.
